# Mechanical thrombectomy of COVID-19 DVT with congenital heart disease leading to phlegmasia cerulea dolens: a case report

**DOI:** 10.1186/s12872-021-02403-w

**Published:** 2021-12-09

**Authors:** Neema Jamshidi, Weiyi Tan, Dingle Foote, Leigh Reardon, Gentian Lluri, Jamil Aboulhosn, John Moriarty, Jeannette Lin

**Affiliations:** 1 Department of Radiological Sciences, UCLA, 757 Westwood Ave, Ste 2125, Los Angeles, CA 90095 USA; 2 Department of Internal Medicine/Division of Cardiology, UCLA, 757 Westwood Ave, Ste 2125, Los Angeles, CA 90095 USA

**Keywords:** Femoral and iliac vein thrombosis, Venous thromboembolism, Mechanical thrombectomy, ClotTriever, Fontan, Case report

## Abstract

**Background:**

COVID-19 and Fontan physiology have each been associated with an elevated risk of venous thromboembolism (VTE), however little is known about the risks and potential consequences of having both.

**Case presentation:**

A 51 year old male with tricuspid atresia status post Fontan and extracardiac Glenn shunt, atrial flutter, and sinus sick syndrome presented with phlegmasia cerulea dolens (PCD) of the left lower extremity in spite of supratherapeutic INR in the context of symptomatic COVID-10 pneumonia. He was treated with single session, catheter directed mechanical thrombectomy that was well-tolerated.

**Conclusions:**

This report of acute PCD despite therapeutic anticoagulation with a Vitamin K antagonist, managed with emergent mechanical thrombectomy, calls to attention the importance of altered flow dynamics in COVID positive patients with Fontan circulation that may compound these independent risk factors for developing deep venous thrombosis with the potential for even higher morbidity.

## Background

COVID-associated coagulopathy has recently been described [1] and COVID VTE is recognized to be significantly elevated in critically ill patients [2–4]. Patients with altered Fontan physiology have independently elevated risks of developing VTE due to decreased pulmonary arterial pressures. Meta-analyses are now reporting risk factors for DVT development including hypoalbuminemia and higher Sequential Organ Failure Assessment (SOFA) scores [5]. However the risk for VTE in the context of altered cardiopulmonary circulation due to congential heart disease (CHD) remains largely unknown.

## Case presentation

**Fig. 1:**
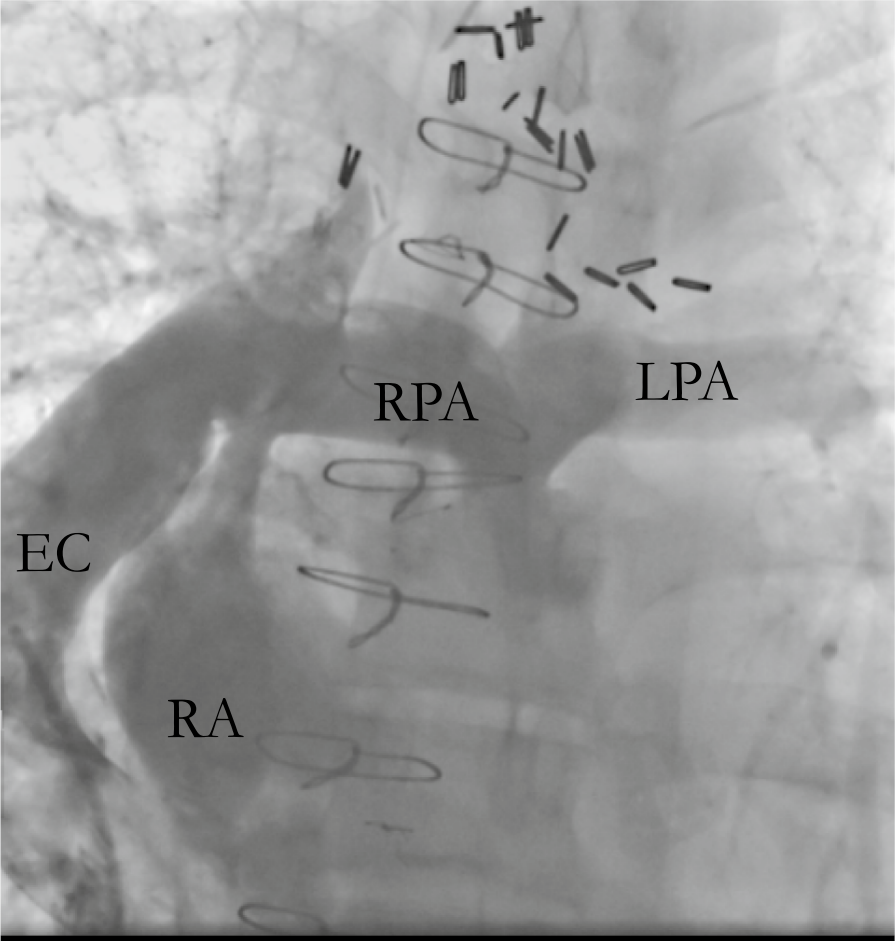
Central venogram highlighting Fontan circulation with extracardiac shunt. *EC* extracardiac Fontan conduit, *RA* right atrium, *RPA* right pulmonary artery, *LPA* left pulmonary artery

**Fig. 2:**
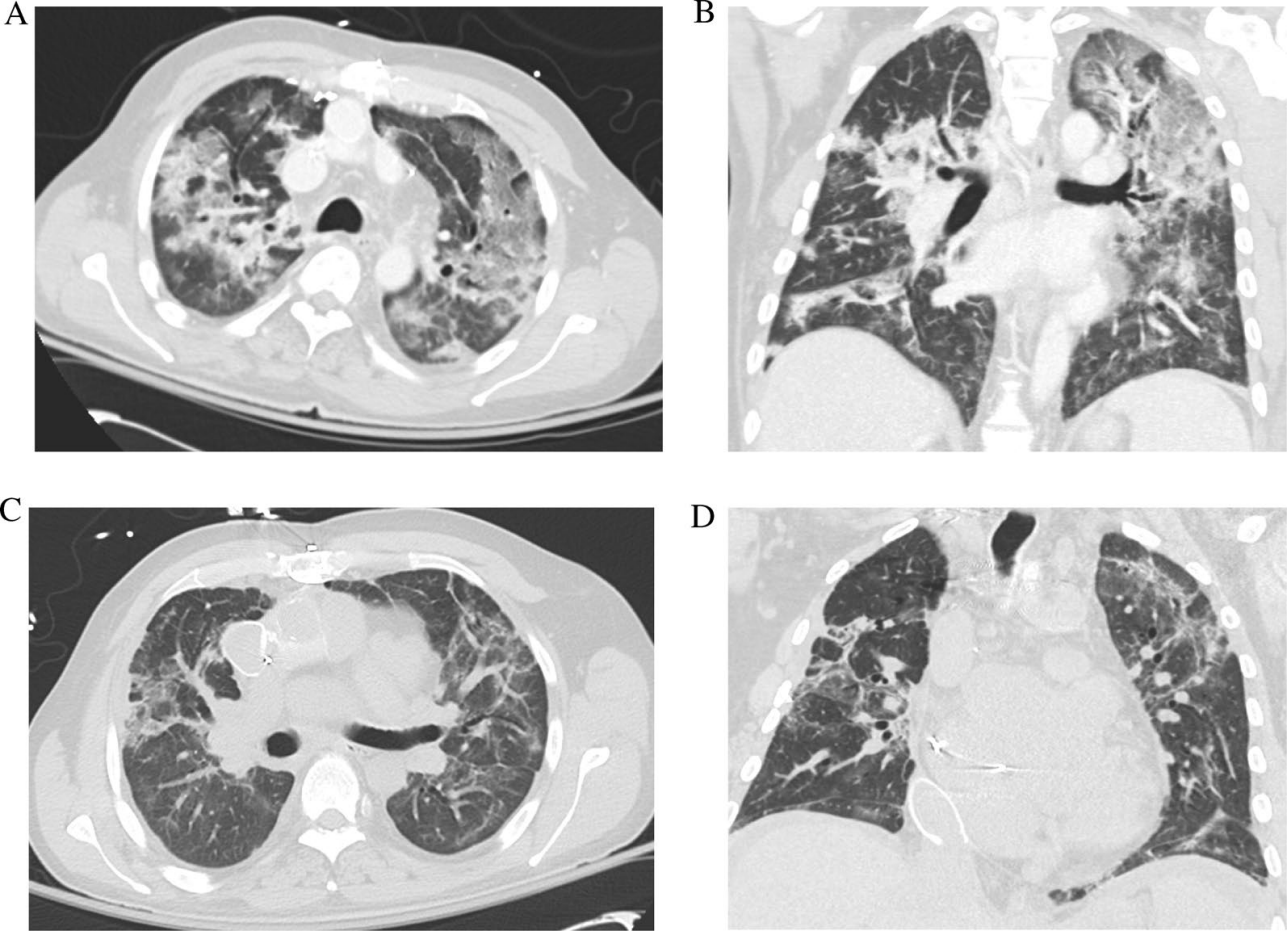
Multifocal pneumonia consistent with clinical presentation and laboratory studies for COVID-19 (**A**, **B**). Subsequent axial and coronal slices showing resolution of COVID-19 pneumonia with pulmonary fibrosis (**C**, **D**)

A 51-year-old male with tricuspid atresia, status post atriopulmonary Fontan at 6 years of age with subsequent urgent surgical conversion to an extracardiac Fontan (Fig. 1) in the setting of a right atrial thrombus at 30 years of age. The patient initially presented to an outpatient clinic with cough and diarrhea. He was diagnosed with SARS-CoV-2 infection via nasopharyngeal swab. Six days later, he presented to a rural emergency room with progressive exertional dyspnea, and was found to be hypoxic and hypotensive. He was diagnosed with COVID-19 pneumonia (Fig. 2), the patient was placed on bilevel positive airway pressure (BiPAP) and vasopressor support. He received a course of remdesivir and was continued on tadalafil. Warfarin was held due to a supratherapeutic international normalized ratio (INR) greater than 14. His respiratory status improved and he was weaned to a non-rebreather mask. He developed bilateral leg pain during the hospitalization, and lower extremity venous ultrasound detected bilateral deep vein thromboses. Given these findings, he was urgently transferred to our institution on hospital day (HD) 7 for higher level of care (Table 1).

**Table 1:** Timeline for the key events from initial presentation, hospitalization, and transfer to our institution

Day 1	Patient presentation to outpatient clinic with upper respiratory and gastrointestinal symptoms, diagnosed with SARS-CoV-2 infection via nasopharyngeal swab
Day 7 (OSH HD 1)	Presentation to community hospital with hypoxia and hypotension with COVID-19 pneumonia requiring BiPAP and vasopressor support
Day 12 (HD 1)	Patient transfered to our healthcare facility for higher level of care with subsequent mechanical thrombectomy of bilateral lower extremities
Day 13	Bilateral iliac filters retrieved with patent IVC and iliac veins
Day 41	Left below the knee amputation was performed
Day 50	Patient discharged to rehabilitation facility

*OSHD* Outside Hospital Day, *HD* Hospital Day (our institution)

**Fig. 3:**
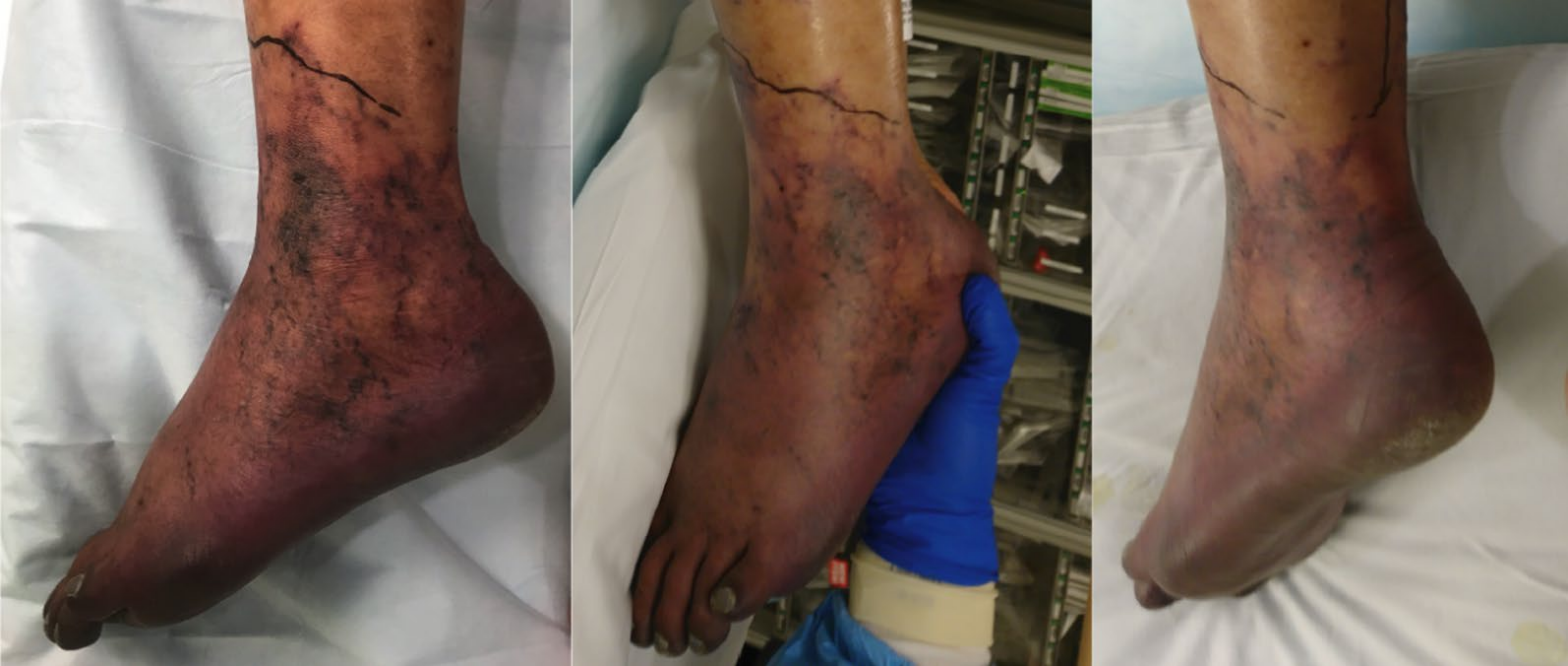
Painful, violaceous foot, concerning for venous outflow obstruction resulting in PCD

**Fig. 4:**
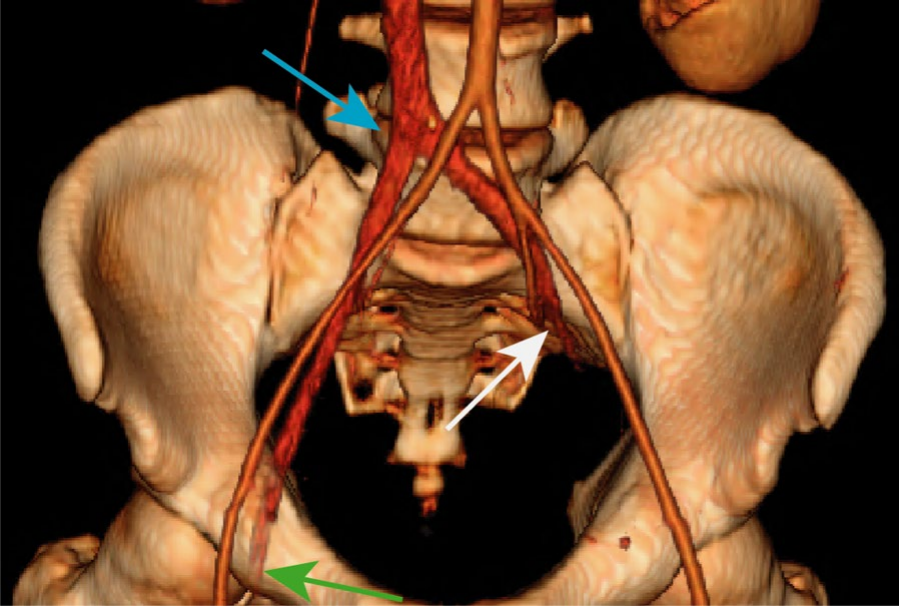
Color-rendered 3D reconstruction of the pelvis outlining the patent arterial vasculature (aorta and bilateral iliac arteries) and IVC with bilateral venous thromboses. The white, green, and blue arrows indicate the abrupt completely occlusive thrombus of the proximal left common iliac vein, the occlusive thrombus of the right common femoral vein, and the iliac confluence into the IVC, respectively

Upon transfer, his INR was down-trending, but remained supratherapeutic at 6.6, and computed tomography venogram (CTV) demonstrated bilateral deep venous thrombosis from the iliac to calf veins, excluding the inferior vena cava (IVC). There was no evidence of pulmonary embolus. Physical exam was notable left lower leg findings pitting edema, purple discoloration (Figs. 3, 4), decreased sensation, and mobility. The laboratory workup was notable in particular for the supratherapeutic INR that was reduced to 1.6 by the second day of hospitalization at our institution, following treatment with Vitamin K. DIC panel and prothrombotic workup, including β-2 glycoprotein and cardiolipin antibodies, were negative (Table 2).

**Table 2:** Relevant laboratory values on admission (Day 12/HD 1)

	Value	Normal range
White blood cell count	8.17	4.16–9.95 × 10E3/uL
Red blood cell count	3.22	4.41–5.95 × 10E6/uL
Hemoglobin	11.6	13.5–17.1 g/dL
Hematocrit	35.7	38.5–52.0%
Mean corpuscular volume	91.5	79.3–98.6 fL
Mean corpuscular hemoglobin	30.7	26.4–33.4 pg
MCH concentration	33.3	31.5–35.5 g/dL
Platelet count	137	143–398 × 10E3/uL
Ferritin	522	8–350 ng/mL
Prothrombin time	16.7	11.5–14.4 s
INR	6.6	< 1.1
APTT	78.5	24.4–36.2 s
D-dimer	5,164	≤ 499 ng/mL FEU
Factor V (5) activity	113	> 50% act
Fibrinogen	526	235–490 mg/dL
Sodium	138	135–146 mmol/L
Potassium	4.1	3.6–5.3 mmol/L
Chloride	101	96–106 mmol/L
Total CO2	25	20–30 mmol/L
Anion gap	12	8–19 mmol/L
Glucose	115	65–99 mg/dL
GFR estimate for non-African American	> 89	> 89 mL/min/1.73m2
Creatinine	0.96	0.60–1.30 mg/dL
Urea Nitrogen	24	7–22 mg/dL
Calcium	7.6	8.6–10.4 mg/dL
Magnesium	1.9	1.4–1.9 mEq/L
Phosphorus	2.7	2.3–4.4 mg/dL
Phosphorus	2.7	2.3–4.4 mg/dL
Lactate dehydrogenase	336	125–256 U/L
Total protein	5.8	6.1–8.2 g/dL
Albumin	2.2	3.9–5.0 g/dL
Bilirubin, total	3.2	0.1–1.2 mg/dL
Bilirubin, conjugated	2.6	≤ 0.3 mg/dL
Alkaline phosphatase	63	37–113 U/L
Aspartate aminotransferase	32	13–47 U/L
Alanine aminotransferase	11	8–64 U/L

While the differential diagnosis of limb ischemia in the setting of COVID-19 infection includes arterial thrombosis, the demonstration of venous outflow obstruction (Fig. 4), violaceous discoloration of the lower extremity, pronounced edema, and pain confirmed the diagnosis of phlegmasia cerulea dolens (PCD). A heparin drip was initiated with vitamin K reversal of warfarin. Since his partial thromboplastin time (PTT) values were abnormal (Table 2), anti-Xa levels were used to titrate the heparin infusion. He also received convalescent plasma [6]. Arterial Doppler was notable for biphasic waveforms with excessive diastolic reversed flow concerning for developing compartment syndrome, without evidence of arterial thrombosis.

Following multidisciplinary evaluation, the decision was made undergo an emergent transvenous thrombectomy to salvage the left leg. Given the inflammatory nature of the clot and development of PCD in spite of supratherapeutic INR, mechanical thrombectomy was felt to be the most appropriate course of action [7] as opposed to ‘lyse and wait’ strategies that could prolong vascular compromise and also subject the patient to increased adverse bleeding events and the potential development of compartment syndrome.

**Fig. 5:**
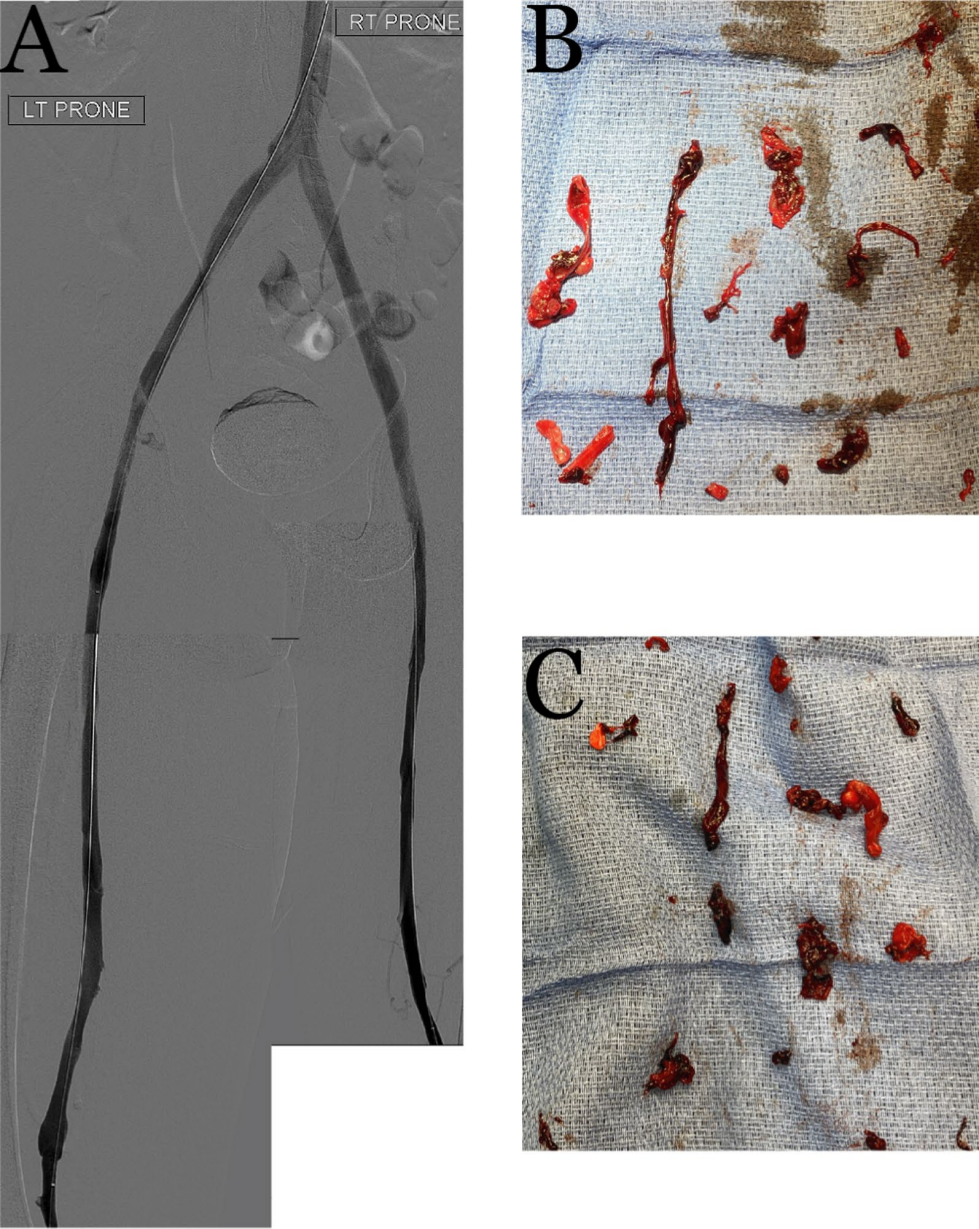
Post-thrombectomy lower extremity venograms (**A**) with corresponding photographs of the pathology-confirmed COVID-19-positive extracted thrombus from the left (**B**) and right (**C**) lower extremities

Bilateral popliteal vein access was obtained for catheter directed mechanical thrombectomy using the ClotTriever® system (Inari Medical). Popliteal and Fontan pressures were 19 and 17 mmHg, respectively; pulmonary angiography did not demonstrate any emboli. Five thrombectomy passes of the left lower extremity from the popliteal vein to the IVC and four passes of the right leg successfully restored flow from the bilateral popliteal veins to the IVC (Fig. 5) and yielded thrombus that was positive for SARS-CoV-2 by polymerase chain reaction (PCR). In order to protect the cardiopulmonary circulation peri-procedurally during anticoagulation optimization, bilateral retrievable iliac vein filters (Optease) were placed. An IVC filter was not placed due to the large size of the cava. The iliac filters were retrieved on HD 12 via femoral approach (the right internal jugular vein was chronically occluded) following venograms confirming patent femoral and iliac veins. A left lower extremity cellulitis developed and empirically managed with antibiotics. The patient unfortunately did not have reversal of the ischemic changes post-procedure, requiring below-the-knee amputation (BKA) for dry gangrene of the left foot.

He experienced epistaxis while on heparin, that was treated with nasal packing. There was evidence of post-pneumonia fibrosis [8] (Fig. 2C and 2D); the associated increase in pulmonary vascular resistance is often not well tolerated in Fontan patients [9]. His tadalafil was increased to 40 mg daily from 20 mg daily to improve hemodynamics. His oxygen saturation was eventually maintained on room air and he never required mechanical ventilation. The BKA was well tolerated and on HD 50 he was discharged to an acute rehabilitation unit on warfarin.

## Discussion and conclusions

Patients with a Fontan circulation are at higher risk for thrombus formation [10]. The systemic venous return drains directly into the pulmonary arteries, and the lack of a subpulmonary ventricle results in a low-flow state through the pulmonary arterial circuit. Pulmonary venous return thus depends on negative inspiratory intrathoracic pressure, low pulmonary vascular resistance, and diastolic function of the systemic ventricle. Increases in pulmonary vascular resistance due to pulmonary infections, positive-pressure ventilation, and/or atrial arrhythmias can all decrease pulmonary blood flow [10, 11]. Patients with COVID-19 infection are at higher risk for thrombosis due to multiple theorized factors, including an increased inflammatory state and endothelial cell dysfunction secondary to viral invasion [12, 13]. PCD in patients with COVID-19 has been reported [1], but thus far only in patients predisposed to thrombosis. In our case, our patient’s cardiac anatomy and physiology placed him in a higher risk category, and despite treatment with a vitamin K antagonist with supratherapeutic levels, he developed extensive bilateral venous thromboses. Interestingly his pulmonary circulation was intact and he did not develop any evidence of pulmonary emboli on CTA or pulmonary angiography.

PCD requires aggressive therapy for limb salvage [14], but resource limitations in heavily impacted hospitals during the COVID pandemic may result in delays in diagnosis and treatment. Nevertheless, the treatment this patient received likely prevented further leg ischemia and limited his amputation to below-the-knee instead of above-the-knee, which is associated with improved functionality and long-term outcomes [15].

Presently, the most effective anticoagulation strategy for patients with COVID-19 related thrombosis is unclear. Our patient developed no further thrombosis while on heparin, though notably this change was made after the COVID pneumonia was already improving.

Patients with comorbidities that place them in higher-risk categories for various disease states, such as Fontan circulation, are potentially at higher risk of complications from COVID-19. PCD is a rare but devastating complication, and must be recognized early and treated aggressively; mechanical thrombectomy may be indicated to expedite restoration of venous outflow, particularly when anticoagulation may be contraindicated. Pulmonary fibrosis secondary to COVID-19 may lead to increased pulmonary vascular resistance, which may further complicate the long-term course of Fontan patients in particular. Despite the presence of acute lung injury and extensive deep venous thromboses, our patient did not require mechanical ventilation or develop decompensated heart failure, reflecting a reassuring degree of resilience of this patient’s Fontan circulation in the face of COVID-19 infection.

## Data Availability

The datasets used and/or analysed during the current study available from the corresponding authors on reasonable request.

## Abbreviations

BKA: Below-the-knee amputation
PCD: Phlegmasia cerulea dolens
PTT: Partial thromboplastin time
CTV: Computed tomography venogram
IVC: Inferior vena cava
HD: Hospital day
BiPAP: Bilevel positive airway pressure
VTE: Venous thromboembolism
DIC: Disseminated intravascular coagulation
PCR: Polymerase chain reaction

## References

[1] Morales MH, Leigh CL, Simon EL. COVID-19 infection with extensive thrombosis: a case of phlegmasia cerulea dolens. Am J Emerg Med. 2020;38(9):1978 e1–e3. 10.1016/j.ajem.2020.05.022 PMC7227523 32425319

[2] Middeldorp S, Coppens M, van Haaps TF (2020). Incidence of venous thromboembolism in hospitalized patients with COVID-19. J Thromb Haemost.

[3] Visveswaran GK, Morparia K, Narang S et al. Severe acute respiratory syndrome coronavirus 2 infection and thrombosis: phlegmasia cerulea dolens presenting with venous gangrene in a child. J Pediatr. 2020;226:281–4 e1. 10.1016/j.jpeds.2020.07.032 PMC7357514 32673617

[4] Bamgboje A, Hong J, Mushiyev S, Pekler G (2020). A 61-year-old man with SARS-CoV-2 infection and venous thrombosis presenting with painful swelling and gangrene of the lower limb consistent with phlegmasia cerulea dolens. Am J Case Rep.

[5] Chen S, Zhang D, Zheng T, Yu Y, Jiang J (2021). DVT incidence and risk factors in critically ill patients with COVID-19. J Thromb Thrombolysis..

[6] Li L, Zhang W, Hu Y (2020). Effect of convalescent plasma therapy on time to clinical improvement in patients with severe and life-threatening COVID-19: a randomized clinical trial. JAMA..

[7] Oguzkurt L, Ozkan U, Demirturk OS, Gur S (2011). Endovascular treatment of phlegmasia cerulea dolens with impending venous gangrene: manual aspiration thrombectomy as the first-line thrombus removal method. Cardiovasc Intervent Radiol.

[8] Spagnolo P, Balestro E, Aliberti S (2020). Pulmonary fibrosis secondary to COVID-19: a call to arms?. Lancet Respir Med..

[9] Gewillig M, Brown SC (2016). The Fontan circulation after 45 years: update in physiology. Heart.

[10] Marrone C, Galasso G, Piccolo R (2011). Antiplatelet versus anticoagulation therapy after extracardiac conduit Fontan: a systematic review and meta-analysis. Pediatr Cardiol.

[11] Fredenburg TB, Johnson TR, Cohen MD (2011). The Fontan procedure: anatomy, complications, and manifestations of failure. Radiographics.

[12] Connors JM, Levy JH (2020). COVID-19 and its implications for thrombosis and anticoagulation. Blood.

[13] Mondal S, Quintili AL, Karamchandani K, Bose S (2020). Thromboembolic disease in COVID-19 patients: a brief narrative review. J Intensive Care.

[14] Mumoli N, Invernizzi C, Luschi R, Carmignani G, Camaiti A, Cei M (2012). Phlegmasia cerulea dolens. Circulation.

[15] Hagberg E, Berlin OK, Renstrom P (1992). Function after through-knee compared with below-knee and above-knee amputation. Prosthet Orthot Int.

